# Optimizing Recovery in Oral Flap Surgeries: The Undervalued Role of Physiotherapy

**DOI:** 10.5041/RMMJ.10552

**Published:** 2025-07-31

**Authors:** Srivatsan Munusamy, Shenbaga Sundaram Subramanian, Tamil Ponni Sivamani, Surya Vishnuram

**Affiliations:** Saveetha College of Physiotherapy (SCPT), Saveetha Institute of Medical and Technical Sciences, Chennai, Tamil Nadu, India

**Keywords:** Oral flap surgery, oral rehabilitation, physiotherapy, post-surgical rehabilitation

**To the Editor**,

We read with great interest the article titled “Local Flap Reconstructions in Oral Cavity Defects: An Insight from 104 Cases” and commend the authors for their valuable contribution to the field of reconstructive surgery.^1^ The study spotlights the importance of local flap reconstructions in oral cancer rehabilitation by offering vital insights into the surgical methods and results of these procedures. We are grateful that *Rambam Maimonides Medical Journal* published this important study, which is essential information for physicians practicing head and neck oncology.

In India, oral carcinoma is the most prevalent cancer in men. Resections used in treatment can result in complicated flaws that significantly alter functional and cosmetic outcomes and lower a patient’s quality of life.^1^ Oral squamous cell carcinoma accounts for 95% of head and neck squamous cell carcinoma cases, making it one of the primary causes of cancer-related deaths globally.^2^ The primary treatment for cancer of the oral cavity is still composite oral resection, neck dissection, and reconstruction using a local, regional, or free flap. Surgery for oral cancer itself, or flap reconstruction, may result in complications. Individuals with certain risk factors, including external variables like radiation therapy, tumor-related factors, and patient-related comorbidities, have greater risks of complications.^3^,^4^ Oral cancers and their surgical management present multiple complications, including impaired oral function, dysphagia, muscle atrophy, trismus, and scar contractures. These issues can significantly affect nutritional intake, speech clarity, and general health in addition to functional impairment. Trismus is considered one of the most challenging postoperative complications, often resulting from fibrosis and scar tissue formation. It can significantly affect a patient’s recovery, with both psychological and functional consequences.^5^ Surgery may not completely solve those problems; thus, additional therapeutic approaches are essential.^5^ A multifaceted approach that goes beyond surgical intervention is essential to address these issues.

Prehabilitation has been demonstrated to increase muscle strength, improve circulation, and prepare tissues for the stress of reconstruction by incorporating specific orofacial and respiratory exercises prior to surgery.^6^ Early physical therapy procedures have been shown to limit fibrosis, enhance oral mobility and speech function, and considerably reduce postoperative sequelae. Orofacial muscle strengthening exercises also improve blood flow and lower postoperative discomfort, thus speeding up the healing process. Additionally, it has been discovered that a multiphasic exercise regimen maximizing surgical patient preparedness improves long-term results. Myofascial release, controlled breathing, and jaw mobility exercises are all components of prehabilitation protocols shown to help patients prepare for major reconstructive surgery.^6^

The clinical significance of such a regimen rests in its ability to enhance postoperative recovery, quality of life, and functional outcomes. Structured physiotherapy protocols typically include exercises for mouth opening, cervical mobilization, active shoulder range of motion, and techniques to improve ventilation and chest mobility, thereby reducing the risk of secretion buildup and pulmonary infection.^7^ Furthermore, secondary complications like orofacial fibrosis, trismus, and speech impairments, which are common following surgery and radiation treatments, can be reduced with intensive physiotherapy. Patients undergoing rehabilitation often demonstrate improvements in tongue mobility, mastication, and general oral function, underscoring the necessity of a systematic and intensive physiotherapy approach.^8^

Although physiotherapy has been shown to be beneficial in the rehabilitation of patients with head and neck cancer, its use varies throughout differing clinical settings. Resistance training, electrical stimulation, and massage therapy are all components of orofacial therapy, which has shown promise in re-establishing facial muscle tone and preventing chronic dysfunction.^9^ Developing an individualized rehabilitation regimen that is suited to each patient’s needs requires a multidisciplinary approach comprising physiotherapists, speech therapists, and oncologists. The role of physical therapy in oral reconstructive surgery is still underutilized in hospital settings, despite increasing evidence of its usefulness.^10^ Standardized rehabilitation procedures and their long-term effectiveness in enhancing functional and cognitive outcomes following flap reconstruction should certainly be the subject of future studies.

In conclusion, we firmly believe that individuals diagnosed with oral cancer undergoing flap reconstruction should have physical therapy incorporated into their prehabilitation and postoperative rehabilitation. Structured physiotherapy interventions can markedly reduce morbidity while improving patient outcomes and general quality of life.
